# Walter Marget and a brief history of paediatric infectious diseases in Munich, Germany

**DOI:** 10.1007/s15010-023-02016-9

**Published:** 2023-04-04

**Authors:** Ulrich von Both, Dieter Adam, Johannes Hübner

**Affiliations:** Department of Infectious Diseases, Dr von Hauner Children’s Hospital, LMU University Hospital, Ludwig Maximilians University, Lindwurmstrasse 4, 80337 Munich, Germany

**Keywords:** Paediatric infectious diseases, INFECTION, Munich, DGI, Paediatrics, Microbiological diagnostics

## Abstract

Theodor Escherich (1857–1911) was one of the key players in early paediatric infectious diseases (PID). In fact, he can be regarded as the first paediatric infectious diseases physician and the founder of this subspecialty. During his long years in service for children, he spent 6 years at the Dr von Hauner children’s hospital (1884–1890), laying the foundations for PID clinical care and research in Munich. Walter Marget, founder of this journal and co-founder of the German Society for Infectious Diseases (DGI) graduated from medical school in 1946 and practised in Munich since 1967. His tireless efforts went into establishing close links between clinical paediatrics and microbiological diagnostics culminating in the foundation of the Department of Antimicrobial Therapy and Infection Epidemiology at the Dr von Hauner children’s hospital. Walter Marget was a key figure for PID in Germany having trained and supported many clinician scientists who followed in his footsteps. This article gives a brief overview of the history of PID in Munich while commemorating Walter Marget and his achievements in this field and for INFECTION.

Looking back in time and narrowing your view on the Dr von Hauner children’s hospital (founded in 1846 by August von Hauner, 1811–1884) at today’s university hospital at the Ludwig-Maximilians-University (LMU) in Munich one can sketch a brief history of paediatric infectious diseases. This story inevitably begins with one of the key players in early infectious disease research, Theodor Escherich. He, according to a recent publication, can be regarded as the first paediatric infectious diseases physician and the founder of this subspecialty [1, 2]. Born in Ansbach, Germany, in 1857, Escherich started his medical studies in 1876 in Wurzburg, continuing in other medical centres including Strasburg, Berlin, Kiel and subsequently Munich. In 1881, he qualified from his medical studies and became very interested in paediatrics serving as first assistant to Professor Karl C. A. J. Gerhardt (1833–1902). Under Gerhardt's supervision he completed his doctoral thesis in Wurzburg (on “Marantic Thrombosinusitis in Cholera Infantum”). He met with Rudolf Emmerich from Munich in 1884 and spent 2 weeks as his scientific assistant investigating the large cholera epidemic in Naples, Italy. He used his newly acquired knowledge in bacteriology to find Vibrio cholerae in stool samples of victims of this epidemic. Since the discipline of paediatrics was yet in its infant stages in Germany at that time, Escherich travelled to Vienna, Austria, to learn from the famous Hermann von Widerhofer (1832–1901), the first Chair of Pediatrics at the University of Vienna, today considered to have been the first “Ordinarius” of the Diseases of Childhood. During this time, he decided to devote his career to child health and began his first bacteriologic investigations of the intestinal microbiological flora of neonates as well as breast milk samples—of what can be today considered as the beginning of microbiome research.

From 1884 he spent a full 6 years at the Dr von Hauner children’s hospital. During these years, he obtained his habilitation [the post-doctoral qualification and licence to lecture in Germany’s universities]. His main area of research covered the normal bacterial flora of the neonatal gastrointestinal tract and its changes after birth, the physiology of digestion and the role of bacteria involved and, finally, characterization of the interdependence of these findings to neonatal disease. Escherich developed close collaborative bonds with the Max von Pettenkofer hygienic institute and Otto Bollinger’s microbiology laboratory where he got more and more familiar with the developing techniques of bacteriology specializing in the characterization of microorganisms. Eventually he isolated almost 20 different bacteria from infant stool samples including various bacilli and cocci. Notably the most important outcome of his works can be considered the detailed descriptions of what he called the *Bacterium coli* commune (today known as *Escherichia coli*) and the *Bacterium lactis* aerogenes (known to us as *Klebsiella pneumoniae*). He was the first paediatrician who took a sincere research and clinical interest in infectious diseases and the study of the normal gastrointestinal flora, today called the gut “microbiome”. In July 1885, Escherich presented the results of his studies on the *Bacterium coli* commune to the Society of Morphology and Physiology in Munich. In 1886 his habilitation thesis “The Intestinal Bacteria of the Infant and Their Relation to the Physiology of Digestion” was published [3]. An English version of this manuscript was reprinted in Reviews of Infectious Diseases in 1988 and 1989 [4, 5].

Escherich himself might have been very impressed by the pivotal role of *E. coli* as prototypical bacterium used in the development of modern molecular biology. At least 10 Nobel prizes have been awarded to scientists for their work on different aspects of *E. coli* biology [1].

Based on these studies and his dedicated service to children and young people, he was regarded as the foremost expert in bacteriology in paediatrics [2]. From 1887, he was a lecturer in Paediatric at the Ludwig-Maximilians-University in Munich, balancing clinical and scientific activities with amazing accuracy and dedication. In 1890, Escherich moved to Austria where he continued his studies and clinical activities in Graz (1890–1902) and Vienna (1902–1911) where he succeeded Hermann von Widerhofer as Chair of Paediatrics at the University of Vienna and Director of the St. Anna Children’s Hospital in Vienna, the most prestigious European position in paediatrics at that time. Those years were spent with a great variety of activities where Escherich demonstrated extraordinary leadership, vision and enthusiasm mirrored in structural developments and modernization as well as the establishment of key services such as the training of nurses in infant care and in providing nursing care to children with tuberculosis or founding the infant welfare society (Säuglingsschutz) as well as the foundation of several scientific societies and the construction of the Imperial Institute for Maternal and Infant Care supported by Emperor Franz Joseph of Austria. It is fair to state that he was well ahead of his time, a true pioneer, in continuously working towards raising awareness about social welfare of children, thus being a great example on multiple levels for us today.

Being an outstanding teacher, clinician and researcher Escherich attracted many brilliant and talented students, such as Meinhard von Pfaundler (1872–1947) or Ernst Moro (1874–1951) who both practised in Munich at the Ludwig-Maximilians-University (LMU) from 1907 and 1906, respectively. He continued to serve the welfare of children until he eventually passed away in Vienna in 1911.

Nine years later, Walter Marget (1920–2013) was born in Stuttgart, Germany. He began his medical studies in 1939 with periods spent in Innsbruck, Vienna and Heidelberg. Due to the turmoil of World War II, it took until 1946 until he was able to complete his medical studies and to start a specialisation in microbiology and hospital hygiene in Heidelberg, culminating in his medical thesis in 1951. His key interest in clinical paediatrics led him to the university children’s hospital in Freiburg where he specialised in paediatrics and put an enormous effort into the establishment of an in-house microbiology laboratory focussed on paediatric infectious diseases. He was a pioneer in this area and the very laboratory still exists today. This activity of structurally and educationally combining clinical paediatrics and microbiology can be highlighted as a hallmark of his professional activity throughout his active years.

His dedicated scientific work focussed on the exploration of nosocomial *Staphylococcus* *aureus* infections in neonatal units with a specific focus on infection control and the thorough investigation and elucidation of transmission events. Making use of the relatively new method of lysotyping, the characterization of different bacterial species by bacteriophage standards [6], he was one of the very first clinical researchers to publish evidence on the reservoir of those pathogens as well as the line of transmission events, results that he published in his habilitation in 1961. In 1962, Marget followed Prof Klaus Betke to Tubingen where he also established a microbiology laboratory in the Children´s hospital, and subsequently moved to Munich in 1967, following Prof. Betke who was appointed as Physician in Chief at the Dr von Hauner Children´s Hospital. Here again, his effort went into establishing close links between clinical paediatrics and microbiological diagnostics mirrored in the establishment of a diagnostic microbiological laboratory and the foundation of the Department of Antimicrobial Therapy and Infection Epidemiology, still existing today. One of Marget’s particular academic interests in later life were the mechanisms of host-defence and specific role that endotoxins, the lipopolysaccharides of Gram-negative bacteria, play in causing clinical disease. His multiple research activities are demonstrated in hundreds of scientific publications and many contributions to medical text books.

Besides his various clinical and academic activities Walter Marget was a great teacher and spent much time and effort on education of students and younger clinicians and researchers. In addition, he founded this very scientific journal, INFECTION, in 1973, a jubilee celebrated in this special edition. INFECTION has since succeeded in becoming one of the leading European journals for Infectious Diseases and clinical microbiology, also serving as the official organ of the German Society for Infectious Diseases (DGI, https://www.dgi-net.de/) that Marget co-founded.

Marget’s educational efforts are also mirrored in him being a co-founder of the European Society for Paediatric Infectious Diseases (ESPID, https://www.espid.org) in 1983 in Munich. Today this scientific society has successfully connected researchers from around the world in joint collaborative efforts in both the clinical and scientific area. ESPID today counts a total of > 1600 members and commemorates Walter Marget by holding an educational workshop at each annual meeting called the Walter-Marget-Educational-Workshop. Finally, Marget supervised over a hundred MD / PhD students and numerous post-doctoral works.

The long tradition of paediatric infectious diseases at the Dr von Hauner Children´s hospital is also a testament of Walter Marget being a great mentor. Several of his co-workers were influenced by him and went on to become important members of the Infectious Disease community in Germany, reflecting in their chosen fields the wide-ranging interests and curiosity of Marget. The following paragraphs as well as Table 1 intend to acknowledge a selection of them.

**Table 1:** Chronology of paediatric infectious disease physicians at the Dr von Hauner Children’s Hospital and themes related to their work

Name	Themes/focus
Theodor Escherich (1857–1911)	Neonatal infections, Infant death, Microbiome (gut)
	Bacterium coli commune (*E. coli*)*, *Children’s welfare
Walter Marget (1920–2013)	Clinical microbiology in paediatrics
	Bacteriology laboratories in children’s hospitals
	Education, Founder of INFECTION, Co-founder of ESPID
Dieter Adam	Clinical pharmacology, therapeutic drug monitoring, co-founder of DGPI and editor of the “DGPI handbook”
Franz Daschner	Infection control and hospital hygiene, hospital epidemiology
Bernd H. Belohradsky	Infection and immunity, primary and acquired immunodeficiencies
Johannes Huebner	Antibiotic Stewardship, antimicrobial resistance, *enterococci*, vaccine development
Ulrich von Both	Antibiotic Stewardship, antimicrobial resistance, paediatric tuberculosis, Refugee & Migrant Health

Dieter Adam studied medicine and pharmacy and received a doctorate in both fields. From 1983 to 2001 he served as head of the Department of Antimicrobial Therapy and Infection Epidemiology at the University Children's Hospital in Munich, succeeding Walter Marget. Adam, who is still an active member of the scientific community today, pioneered pharmacological studies and helped to elucidate pharmacokinetics and pharmacodynamics of antimicrobials. He served as president of the Paul Ehrlich Society for Chemotherapy from 1982 to 1986 and as president of the German Society for Pediatric Infectious Disease (DGPI) from 1993 to 1999.

Franz Daschner, like Dieter Adam an early disciple of Marget’s, worked between 1970 and 1975 at the Dr von Hauner Children´s Hospital. From 1976 onwards, he served as head of the Infection Control Department at the University Hospital of Freiburg and subsequently from 1992 until his retirement in september 2006 as director of the Institute for Environmental Medicine and Hospital Hygiene in Freiburg, founded by himself. Daschner was the first physician to be awarded the German Environmental Prize of the German Federal Environmental Foundation for his important work to assess and minimize the negative environmental effects of hygiene measures in medicine.

Bernd Belohradsky succeeded Dieter Adam as head of the Department of Antimicrobial Therapy and Infection Epidemiology. His clinical and scientific focus, most likely encouraged by Walter Marget, was laid on inborn and acquired immunodeficiencies, an important and newly emerging field at that time. Belohradsky established the Immunodeficiency Outpatient Clinics (Immundefektambulanz, IDA) still an important specialty service in our days for children with rare genetically mediated immunological diseases as well as acquired immunodeficiency, such as an HIV infection.

Currently the legacy of paediatric infectious diseases at the Dr von Hauner children’s hospital is carried on by two authors of this manuscript, Johannes Huebner and Ulrich von Both. One major joint clinical and research area of these clinician scientists revolves around antimicrobial resistance and the implementation of antibiotic stewardship (ABS) interventions. Since 2015, an ABS team consisting of paediatric ID physicians, pharmacists and supporting scientists has been established at the children’s hospital and been actively growing ever since. Until today many junior doctors have been trained in ABS and infectious diseases of childhood following an educational curriculum. Research projects have focussed on aspects of ABS both in the University Hospital and in the outpatient (private paediatric practices) setting. Recently, a national team of infectious diseases specialists led by Huebner and von Both has been awarded a grant to establish an ABS programme to improve medical care and rational antibiotic use in more than 30 non-university paediatric hospitals by means of an innovative telemedicine approach (https://www.tele-kasper.de/tele-kasper). Additional research areas addressed by the current team of paediatric infectious diseases include work on enterococcal infections, phage therapy and vaccine development (Huebner) as well as paediatric tuberculosis, improved biomarkers for viral or bacterial infections and activities in refugee & migrant health (von Both).

Since the time of Theodor Escherich, paediatric infectious disease specialists at the Dr von Hauner Children’s Hospital have been pioneers exploring new areas of infectious diseases research and medical care (Fig. 1). From the first description the *Bacterium coli* commune and the beginning of microbiome research via the implementation of close links between clinical paediatrics and microbiological diagnostics and an in-depth exploration of fundamental principles and their practical implications of hospital hygiene to a current innovative telemedical approach to broaden the scope of antibiotic stewardship service to paediatric patients in Germany—this pioneering journey is ever continuing at the Dr von Hauner Children’s hospital and its PID department once established by the founding father of the INFECTION journal: Walter Marget.

**Fig. 1:**
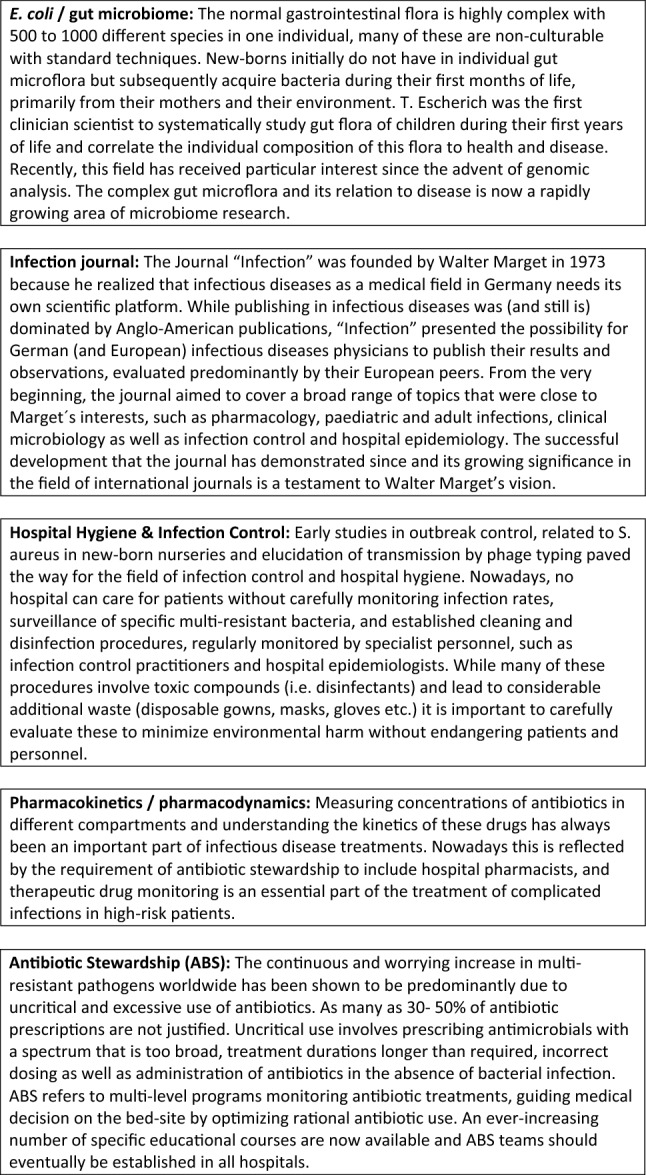
Contributions of Theodor Escherich, Walter Marget and his successors at the Dr von Hauner Children’s Hospital to science and clinical care

## Data Availability

Not Applicable.
